# Production, crystallization and preliminary crystallographic analysis of an exosite-containing fragment of human von Willebrand factor-cleaving proteinase ADAMTS13

**DOI:** 10.1107/S1744309109023410

**Published:** 2009-06-30

**Authors:** Masashi Akiyama, Soichi Takeda, Koichi Kokame, Junichi Takagi, Toshiyuki Miyata

**Affiliations:** a National Cardiovascular Center Research Institute, 5-7-1 Fujishirodai, Suita, Osaka 565-8565, Japan; b Laboratory of Protein Synthesis and Expression, Institute for Protein Research, Osaka University, 3-2 Yamadaoka, Suita, Osaka 565-0871, Japan

**Keywords:** von Willebrand factor-cleaving proteinase, ADAMTS13, metalloproteinases, ancillary domains

## Abstract

ADAMTS13 is a reprolysin-type metalloproteinase belonging to the ADAMTS (a disintegrin and metalloproteinase with thrombospondin type 1 motif) family. It specifically cleaves plasma von Willebrand factor (VWF) and regulates platelet adhesion and aggregation. ADAMTS13 is a multi-domain enzyme. In addition to the N-terminal metalloproteinase domain, the ancillary domains, including a disintegrin-like domain, a thrombospondin-1 type 1 repeat, a Cys-rich domain and a spacer domain, are required for VWF recognition and cleavage. In the present study, a fragment of the ADAMTS13 ancillary domains (ADAMTS13-DTCS; residues 287–685) was expressed using CHO Lec cells, purified and crystallized. Diffraction data sets were collected using the SPring-8 beamline. Two ADAMTS13-DTCS crystals with distinct unit-cell parameters generated data sets to 2.6 and 2.8 Å resolution, respectively.

## Abstract 2

A fragment of the ADAMTS13 ancillary domains (ADAMTS13-DTCS) has been expressed, purified and crystallized and the crystals have been characterized by X-ray diffraction.

## 1. Introduction

von Willebrand factor (VWF) is a plasma glycoprotein that is involved in platelet-dependent haemostasis (Sadler, 1998 ▶). VWF is primarily synthesized in vascular endothelial cells and megakaryo­cytes and is released into the plasma as ultralarge multimeric forms (UL-VWF) that are highly active in platelet adhesion and aggregation. A plasma metalloproteinase, ADAMTS13, specifically cleaves the Tyr1605-Met1606 peptidyl bond within the A2 domain of VWF (Dent *et al.*, 1990 ▶). Cleavage of UL-VWF into smaller forms by ADAMTS13 limits platelet thrombus formation. A deficiency of ADAMTS13 enzymatic activity caused by either genetic mutations in the ADAMTS13 gene or acquired autoantibodies against ADAMTS13 results in the accumulation of UL-VWF in plasma. This leads to the formation of disseminated platelet-rich microthrombi in arterioles, which is one of the characteristic pathogenic features of thrombotic thrombocytopaenic purpura (TTP), a life-threatening systemic disease (Tsai, 2009 ▶). Conversely, excessive cleavage of VWF causes von Willebrand disease type 2A (Sadler, 2005 ▶).

Human ADAMTS13 consists of 1427 amino acids and has a modular structure comprising a signal peptide, a short propeptide, a metalloproteinase domain (M), a disintegrin-like domain (D), a thrombospondin-1 type 1 repeat (T1), a Cys-rich domain (C), a spacer domain (S), seven additional type 1 repeats (T2–T8) and two CUB (C1r/C1s, urinary epidermal growth factor, bone morphogenic protein) domains (Levy *et al.*, 2001 ▶; Soejima *et al.*, 2001 ▶; Zheng *et al.*, 2001 ▶). C-terminal truncation of ADAMTS13 after the C but not the S domain results in severe loss of proteolytic activity towards VWF (Soejima *et al.*, 2003 ▶; Zheng *et al.*, 2003 ▶). Therefore, in addition to the M domain, the ancillary domains including the D, T, C and S domains (DTCS) are necessary for normal ADAMTS13 activity, although the distal C-terminal domains are required for regulation of *in vivo* thrombus formation under high-shear conditions (Banno et al., 2009 ▶). We have previously reported a minimal functional substrate con­sisting of 73 amino-acid residues of the C-terminal region of the VWF A2 domain (Asp1596–Arg1668) and designated VWF73 (Kokame *et al.*, 2004 ▶; Miyata *et al.*, 2007 ▶). A recent study has shown that the VWF-binding exosites located in the T, C and S domains interact with different segments of VWF73 (Gao *et al.*, 2008 ▶). These interactions increased the VWF-binding affinity and rate of substrate cleavage by 300-fold. At least 16 causative missense mutations for congenital TTP and five missense polymorphisms in the ADAMTS13 gene have been identified within the DTCS region (Levy *et al.*, 2001 ▶; Kokame *et al.*, 2002 ▶; Banno & Miyata, 2008 ▶). Although most TTP-causative mutant proteins are likely to show secretion deficiency, a P475S polymorphism variant showed normal secretion but reduced VWF-cleaving activity (Kokame *et al.*, 2002 ▶; Akiyama *et al.*, 2008 ▶). Detailed structural information on exosite-containing domains will help in understanding the structure-based mechanism of substrate recognition and specificity and the effects of TTP-causative mutations and common polymorphisms. To date, crystal structures of the M and D domains of three human ADAMTS-family proteins, ADAMTS1, ADAMTS4 and ADAMTS5, have been reported (Gerhardt *et al.*, 2007 ▶; Mosyak *et al.*, 2008 ▶; Shieh *et al.*, 2008 ▶). However, no crystal structures of exosite-containing fragments of ADAMTSs have been reported.

Here, we report the expression and purification of the exosite-containing human ADAMTS13-DTCS fragment using mammalian CHO Lec cells with mutations in multiple glycosylation-related genes. Proteins obtained from this cell line are suitable for crystallization because their restricted and homogeneous glycosylation improves the packing of the protein molecules. We also report the results of our crystallization and preliminary X-ray studies of ADAMTS13-DTCS.

## 2. Methods

### 2.1. Expression and purification of ADAMTS13-DTCS

**Figure 1:**
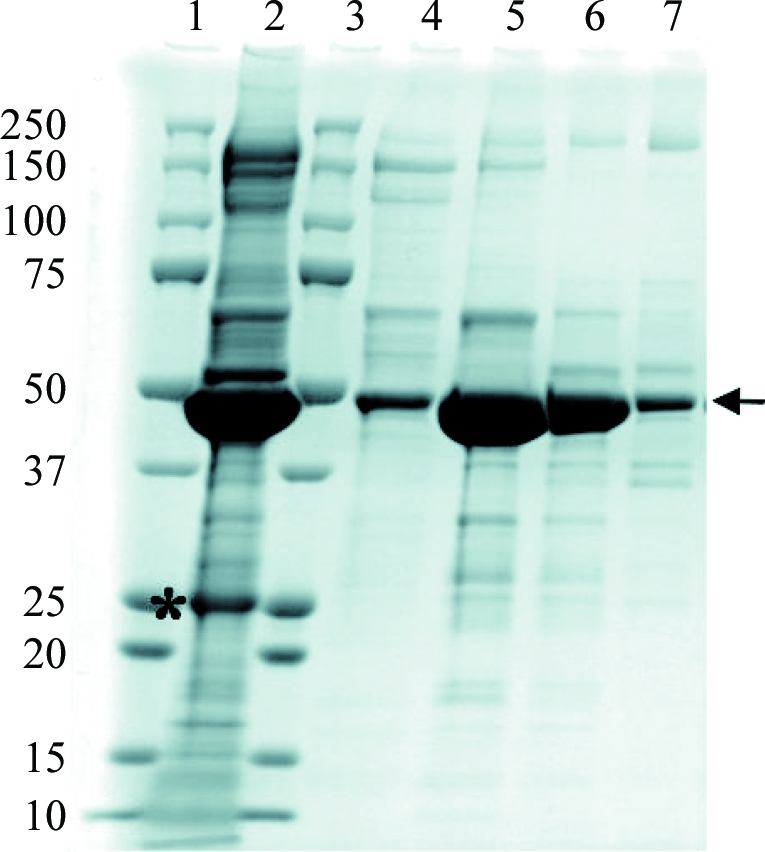
SDS–PAGE analysis of ADAMTS13-DTCS fractions from Hi-Trap SP HP cation chromatography. Proteins were analyzed by SDS–PAGE and stained with Coomassie Brilliant Blue. Lanes 1 and 3, molecular-weight markers (kDa); lane 2, pooled Ni–NTA eluate treated with TEV proteinase; lanes 4–7, eluate fractions Nos. 5–8, respectively, from the Hi-Trap SP HP column. Arrow, ADAMTS13-DTCS. Asterisk, TEV proteinase.

An ADAMTS13 cDNA (AB069698) fragment corresponding to amino-acid residues 287–685 (ADAMTS13-DTCS) was amplified by PCR and cloned into a mammalian expression vector based on pcDNA3.1/Myc-His (Invitrogen), which has a mouse *Nid1* signal sequence (Yasui *et al.*, 2007 ▶). The nucleotide sequence was confirmed by dye-terminator sequencing. The ADAMTS13-DTCS fragment expressed from this vector contains a tobacco etch virus (TEV) proteinase cleavage site (Glu-Asn-Leu-Tyr-Phe-Gln/Gly) followed by tandem His-tag sequences at the C-terminus. We transfected the plasmid into CHO Lec 3.2.8.1 cells (Stanley, 1989 ▶) by electroporation and selected colonies resistant to G418 (3 mg ml^−1^) on 96-well plates in α-minimal essential medium supplemented with 5% foetal bovine serum for 10 d. ADAMTS13-DTCS levels in the media of 48 G418-resistant colonies were examined by Western blotting with anti-6×His antibody (Sigma–Aldrich, St Louis, Missouri, USA). The clone with the highest secretion level of ADAMTS13-DTCS was cultured in the medium containing 0.5 mg ml^−1^ G418 by the roller-bottle method and the medium was collected every 3 d. The ADAMTS13-DTCS was recovered from the culture medium by 50%(*w*/*v*) ammonium sulfate precipitation and was purified by Ni–­NTA agarose chromatography (Sigma–Aldrich). The eluted ADAMTS13-DTCS was incubated with TEV proteinase for 12 h at 297 K to remove the C-­terminal tags. After dialysis in a buffer consisting of 10 m*M* MES and 100 m*M* NaCl pH 6.0, the digest was applied onto a Hi-Trap SP HP cation-exchange column (GE Healthcare, Buckinghamshire, England). The column was washed with the same buffer and ADAMTS13-DTCS was eluted with a linear gradient of NaCl (0.1–0.7 *M*) in 10 m*M* MES pH 6.0. Fractions were analyzed by SDS–PAGE under reducing conditions (Fig. 1 ▶). The fractions rich in ADAMTS13-DTCS (lanes 5 and 6) were combined, dialyzed against 10 m*M* MES pH 6.0 and concentrated using a Vivaspin-5 separation device (30 kDa molecular-weight cutoff; Sartorius, Edgewood, New York, USA) to a final concentration of ∼10 mg ml^−1^ for crystallization.

### 2.2. Crystallization screening

Initial screening for crystallization conditions for ADAMTS-DTCS was carried out by the sitting-drop vapour-diffusion method using Index Screen, SaltRx Screen, PEG/Ion Screen, Grid Screen MPD and Grid Screen Ammonium Sulfate kits (Hampton Research, Aliso Viejo, California, USA). A volume of 0.1 µl protein solution was manually mixed with an equal amount of reservoir solution and the droplets were allowed to equilibrate against 0.1 ml reservoir solution at 293 K for 24 h.

### 2.3. Diffraction data collection

For X-ray measurements, crystals were soaked in a solution containing 20% glycerol, 26% PEG 1500, 100 m*M* MES pH 6.0 for cryoprotection prior to flash-freezing and were immediately exposed to a stream of nitrogen gas at 100 K. Preliminary X-ray data were collected using an in-house X-ray diffractometer (Micromax-007 X-­ray generator with an R-AXIS VII imaging-plate detector; Rigaku, Tokyo, Japan) and diffraction-quality crystals were selected for data acquisition using the SPring-8 beamline. All the diffraction data sets were collected on beamline BL41XU at 100 K using an ADSC Quantum 310R detector and the diffraction images were processed using *HKL*-2000 software (Minor *et al.*, 2006 ▶).

## 3. Results and discussion

### 3.1. Protein preparation

We first attempted to express ADAMTS13-DTCS in *Escherichia coli* and insect cells. The expressed ADAMTS13-DTCS formed inclusion bodies and renaturation of ADAMTS13-DTCS did not succeed. We then tried to express ADAMTS13-DTCS in mammalian cells. As ADAMTS13-DTCS contains four potential N-glycosylation sites, we used the CHO Lec 3.2.8.1 cell line for stable expression. This cell line has four different mutated genes that are involved in the N- and O-­glycosylation pathways (Stanley, 1989 ▶). Preliminary experiments showed that the endogenous signal and propeptide sequences of ADAMTS13 resulted in low protein secretion. We replaced the signal sequence and prosequence with the mouse *Nid1* signal sequence (Mann *et al.*, 1989 ▶). This replacement dramatically increased the secretion of ADAMTS13-DTCS into the medium. ADAMTS13-DTCS was purified by Ni–NTA chromatography followed by Hi-Trap SP cation-exchange chromatography. The molecular weight of the recombinant protein, 45 kDa, estimated by SDS–PAGE coincided well with the estimated molecular weight of 46 kDa (Fig. 1 ▶). Fractions (Fig. 1 ▶, lanes 5 and 6) from the Hi-Trap SP column were combined and used for crystallization without further purification. Approximately 6 mg ADAMTS13-DTCS was recovered from 20 l culture medium.

### 3.2. Crystallization

**Figure 2:**
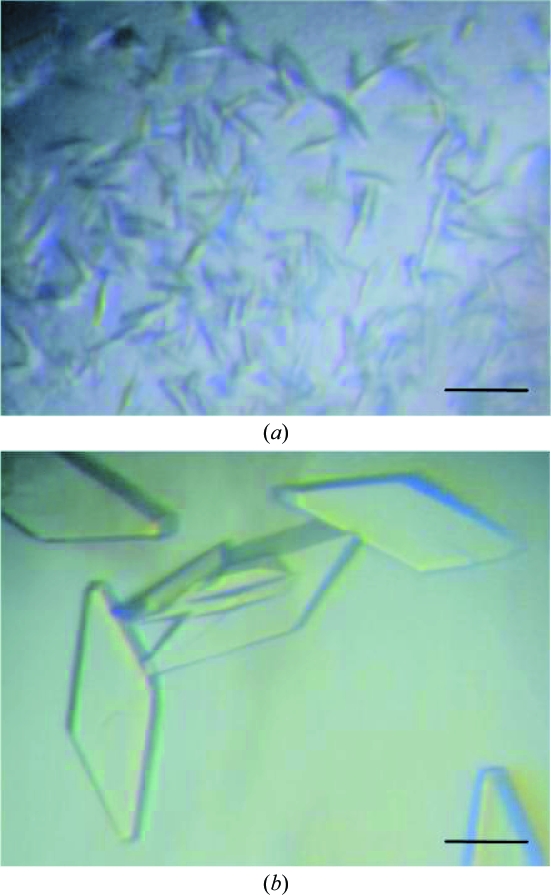
Crystals of human ADAMTS13-DTCS grown by the sitting-drop method. (*a*) Microcrystals obtained from solution No. 4 of the PEG/Ion Screen kit. (*b*) Crystals obtained using the optimized conditions. The scale bars indicate 0.1 mm.

Of the 288 initial crystallization conditions tested, 20 yielded microcrystals (Fig. 2 ▶ *a*). Using solution No. 4 of the PEG/Ion Screen kit [0.2 *M* lithium chloride, 20%(*w*/*v*) PEG 3350 pH 6.8] as a starting condition, the pH of the mother liquor, the concentration and molecular weight of the PEG and the species and concentrations of salts and additives were optimized. The combination of refinement of the crystallization conditions and an increase in the protein concentration (to ∼20 mg ml^−1^) improved the size of the crystals. Single crystals were obtained from drops made up of 0.5 µl protein solution and 0.5 µl reservoir solution [26%(*w*/*v*) PEG 1500, 100 m*M* MES pH 6.0] supplemented with a one-fifth volume of 40%(*w*/*w*) penta­erythritol ethoxylate (3/4 EO/OH; Additive Screen solution No. 52; Hampton Research). Crystals with dimensions of 300 × 100 × 50 µm were formed after 3–7 d at 293 K (Fig. 2 ▶ *b*).

### 3.3. X-ray analysis

All diffraction data sets were acquired using the oscillation method on beamline BL41XU at a wavelength of 1.0 Å. The oscillation angle was 1.0° for all data sets. The native data sets for form 1 and form 2 contained 16 867 (2.60 Å resolution) and 12 811 (2.80 Å resolution) unique reflections, respectively. The asymmetric unit was estimated to contain one molecule, with corresponding crystal volume per protein weights of 2.7 and 3.1 Å^3^ Da^−1^ for crystal forms 1 and 2, respectively. Solvent-content estimations based on a single copy of the molecule per asymmetric unit gave values of 53.4% and 48.6% for crystal forms 1 and 2, respectively. The X-ray data showed that the two crystal forms have different unit-cell parameters even when they are obtained under identical conditions. The variation in crystal packing might reflect the mobility of the domains in ADAMTS13-DTCS. The statistics of the data sets are summarized in Table 1 ▶.

### 3.4. Screening of heavy-atom derivatives

We attempted experimental phasing using heavy-atom derivatives because molecular replacement was not an option owing to the lack of related structures. The limited availability of single large crystals owing to the small amount of ADAMTS13-DTCS and its tendency to form multiple crystals produced difficulties in the search for heavy-atom derivatives. Therefore, we focused on investigating the colouring of crystals on heavy-atom soaking, which can be a good indication of heavy-atom binding. We selected 13 coloured com­pounds (Au-6, M1-­10, M-11, M1-14, M1-15, M1-16, M1-17, M2-2, M2-­3, M2-5, M2-16, M2-17 and M2-18) from Heavy Atom Screens (Hampton Research) and soaked small crystals in reservoir solution supplemented with each of these compounds. After several hours, we found that three osmium-containing compounds, ammonium hexa­bromoosmate (M2-16), potassium hexachloroosmate (M2-17) and osmium chloride (M2-18), were heavily absorbed into the crystals. To examine the X-­ray diffraction from these potential derivatives, we prepared larger single crystals. We checked these using the in-house X-ray facility and well diffracting crystals were shipped for data acquisition at SPring-8. Structural analysis is now in progress using data obtained from the derivative soaked in the osmium chloride solution.

## Figures and Tables

**Table 1:** Data-collection statistics for ADAMTS13-DTCS crystals Values in parentheses are for the highest resolution shell. A single crystal was used for measurement for each data set.

	Form 1	Form 2
Space group	*C*2	*C*2
Unit-cell parameters		
*a* (Å)	152.7	138.6
*b* (Å)	52.9	51.4
*c* (Å)	76.2	76.4
β (°)	111.4	106.7
Wavelength (Å)	1.0	1.0
Resolution (Å)	50–2.60 (2.69–2.60)	30–2.80 (2.90–2.80)
No. of unique reflections	16867 (1272)	12811 (1259)
*R*_merge_†	0.052 (0.176)	0.062 (0.403)
*I*/σ(*I*)	19.3 (5.7)	13.3 (3.5)
Completeness (%)	95.3 (72.7)	99.5 (99.3)
Redundancy	3.5 (2.9)	3.7 (3.6)
Matthews value (Å^3^ Da^−1^)	2.64	2.39
Solvent content (%)	53.4	48.6

† *R*_merge_ = 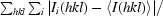 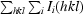, where *I*_*i*_(*hkl*) is the *i*th intensity measurement of reflection *hkl* and 〈*I*(*hkl*)〉 is its weighted average.
